# Growth of human bronchial epithelial cells at an air-liquid interface alters the response to particle exposure

**DOI:** 10.1186/1743-8977-10-25

**Published:** 2013-06-26

**Authors:** Andrew J Ghio, Lisa A Dailey, Joleen M Soukup, Jacqueline Stonehuerner, Judy H Richards, Robert B Devlin

**Affiliations:** 1 The Environmental Public Health Division, NHEERL, USEPA, Research Triangle Park, NC 27711, USA; 2 Human Studies Facility, USEPA, 104 Mason Farm Road, Campus Box 7315, Chapel Hill, NC 27599-7315, USA

**Keywords:** Cell differentiation, Air pollution, Particulate matter, Anoxia

## Abstract

**Background:**

We tested the hypothesis that normal human bronchial epithelial (NHBE) cells 1) grown submerged in media and 2) allowed to differentiate at air-liquid interface (ALI) demonstrate disparities in the response to particle exposure.

**Results:**

Following exposure of submerged NHBE cells to ambient air pollution particle collected in Chapel Hill, NC, RNA for IL-8, IL-6, heme oxygenase 1 (HOX1) and cyclooxygenase 2 (COX2) increased. The same cells allowed to differentiate over 3, 10, and 21 days at ALI demonstrated no such changes following particle exposure. Similarly, BEAS-2B cells grown submerged in media demonstrated a significant increase in IL-8 and HOX1 RNA after exposure to NIST 1648 particle relative to the same cells exposed after growth at ALI. Subsequently, it was not possible to attribute the observed decreases in the response of NHBE cells to differentiation alone since BEAS-2B cells, which do not differentiate, showed similar changes when grown at ALI. With no exposure to particles, differentiation of NHBE cells at ALI over 3 to 21 days demonstrated significant decrements in baseline levels of RNA for the same proteins (i.e. IL-8, IL-6, HOX1, and COX2). With no exposure to particles, BEAS-2B cells grown at ALI showed comparable changes in RNA for IL-8 and HOX1. After the same particle exposure, NHBE cells grown at ALI on a transwell in 95% N_2_-5% CO_2_ and exposed to NIST 1648 particle demonstrated significantly greater changes in IL-8 and HOX1 relative to cells grown in 95% air-5% CO_2_.

**Conclusions:**

We conclude that growth of NHBE cells at ALI is associated with a diminished biological effect following particle exposure relative to cells submerged in media. This decreased response showed an association with increased oxygen availability.

## Background

The airway epithelium provides a critical interface between the body and the external environment. In the human, this epithelium is a pseudostratified layer consisting of basal cells, secretory cells, and columnar ciliated cells [1]. It exerts a protective effect through a number of distinct defense mechanisms including 1) the provision of a physical barrier, 2) secretion of factors that mediate immunity, inflammation, and antioxidant defense, and 3) clearance of materials through a mucociliary pathway. The presence of the airway epithelium appears to be absolutely necessary for host defenses against inhaled particles and microbes [2-4].

When cultured at an air–liquid interface (ALI) in an appropriate medium, normal human bronchial epithelial cells (NHBE) form a polarized, pseudostratified epithelium composed of ciliated and mucus-secreting cells [5,6]. This culture system provides a useful tool for the *in vitro* study of airway epithelial biology and cell differentiation (i.e. increasing specialization leading to the formation of structurally and functionally distinct cells, tissues, and organs). The *in vitro* response of differentiated airway epithelial cells may more accurately predict that of the lung relative to that of submerged cells [7].

Differentiation of NHBE cells occurs only at ALI with an increased cellular availability of O_2_. We tested the hypothesis that relative to submerged cells, NHBE cells grown at ALI and allowed to differentiate would have an altered response to particle exposure.

## Results

**Figure 1:**
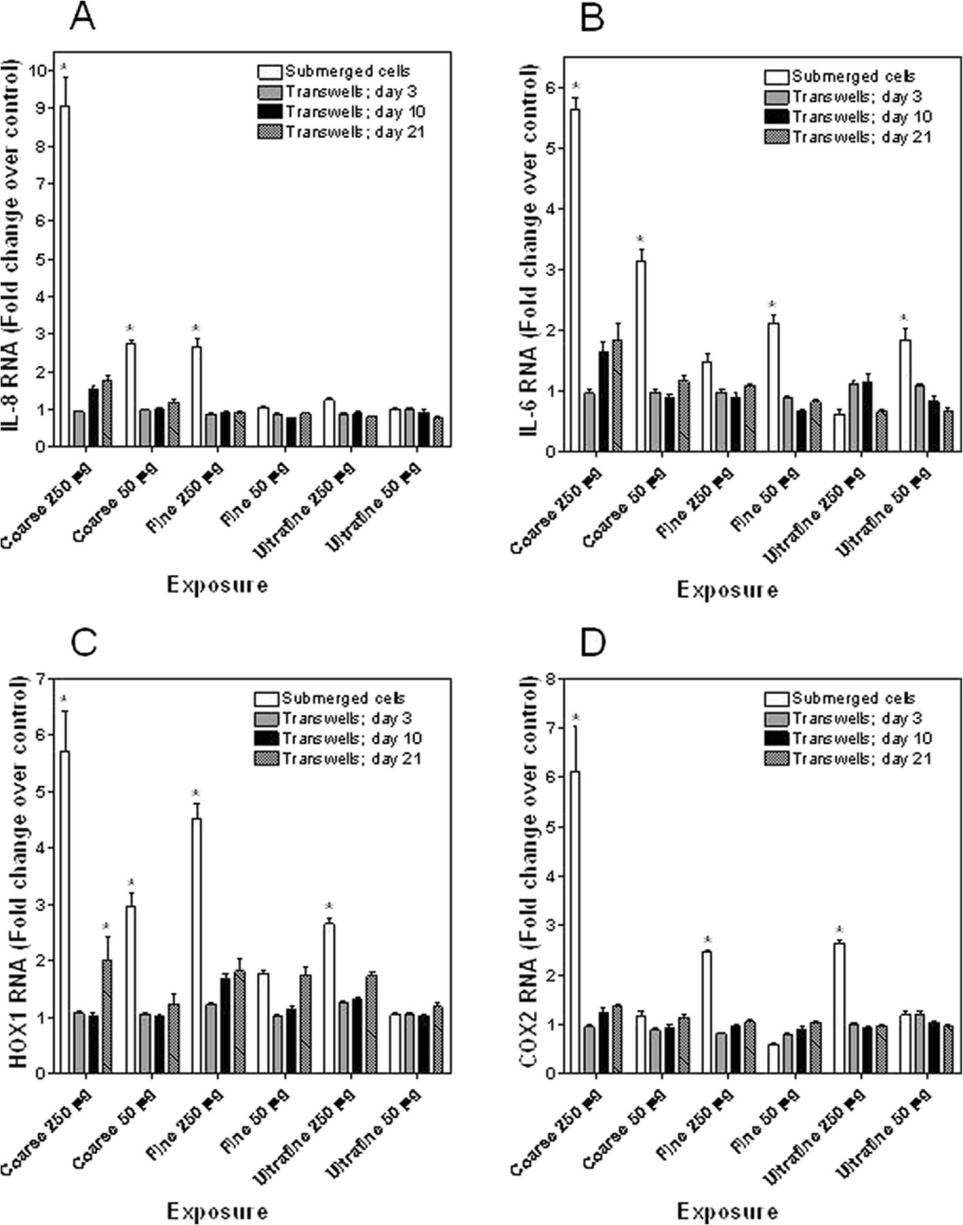
**Fold change RNA of NHBE cells for IL-8 (A), IL-6 (B), HOX1 (C), and COX2 (D) following exposure to fractions of Chapel Hill ambient air pollution particle.** Significant increases in RNA were observed after exposure of submerged cells to the particles fractions. However, no increased RNA was observed following exposure of NHBE cells grown at ALI to the same particle. Data was statistically analyzed using a one way ANOVA only; no effect of either fraction or mass was evaluated. *Significant increase relative to RNA in unexposed NHBE cells.

Relative to submerged cells, NHBE cells grown at ALI for 21 days showed evidence of differentiation with 18.6 ± 3.9 and 11.8 ± 3.0 fold increased RNA for alpha tubulin and muc5b respectively [6]. NHBE cells grown submerged in media demonstrated a significant increase in RNA for the pro-inflammatory mediators IL-8 and IL-6 at 4 hr following exposure to ambient air pollution particle collected from Chapel Hill, North Carolina (Figures 1A and 1B). Elevations in IL-8 and IL-6 RNA were greatest following exposure to the coarse fraction in Chapel Hill particle. NHBE cells allowed to differentiate at ALI demonstrated no elevations in RNA for IL-8 and IL-6 at 4 hr after PM exposure (Figures 1A and 1B). Similarly, there was increased RNA for two proteins involved in oxidative stress at 4 hr following exposure of submerged NHBE cells to particles (Figures 1C and 1D). Those cells which differentiated at ALI did not show such elevations in HOX1 and COX2 after exposure to PM except day 21 transwell cultures exposed to the higher dose of coarse particle (Figures 1C and 1D).

**Figure 2:**
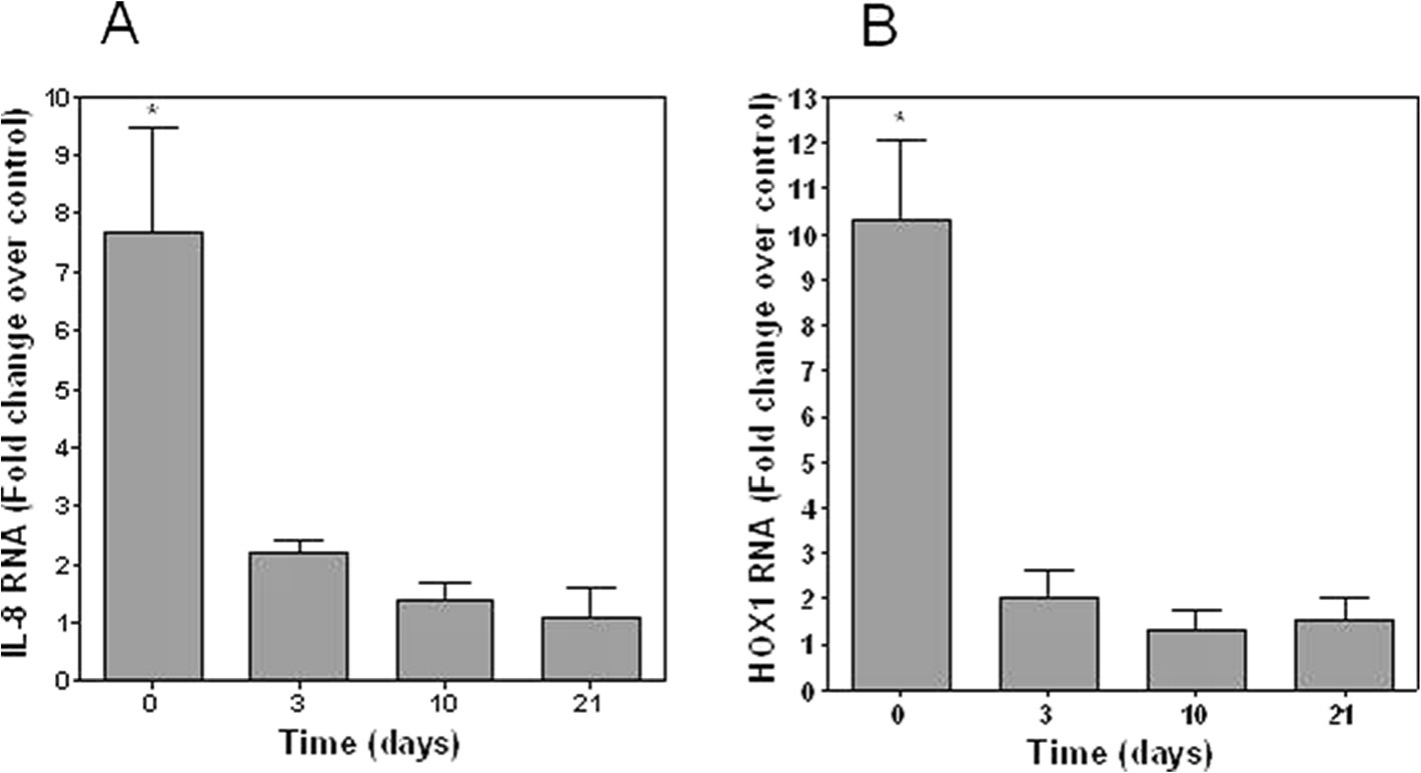
**Fold change RNA of BEAS-2B cells for IL-8 (A) and HOX1 (B) following exposure to 250 μg NIST 1648.** There were significant increases in RNA after exposure of submerged cells only. In contrast, no increased RNA was observed following exposure of BEAS-2B cells grown at ALI to the same particle. *Significant increase relative to RNA in unexposed BEAS-2B cells.

To evaluate the role of differentiation in changes of PM response, BEAS-2B cells were employed; these cells do not differentiate. BEAS-2B cells grown for 21 days on the transwell continued to proliferate. The cells retained a cobblestone appearance but the cellular density increased with heaping or stacking evident. Submerged BEAS-2B cells demonstrated a significant increase in IL-8 and HOX1 RNA after 4 hour exposure to NIST 1648 particle (Figures 2A and 2B). However, the same cells grown at ALI and exposed to the same particle showed no change in IL-8 and HOX1 RNA comparable to the response of the NHBE cells (Figures 2A and 2B).

**Figure 3:**
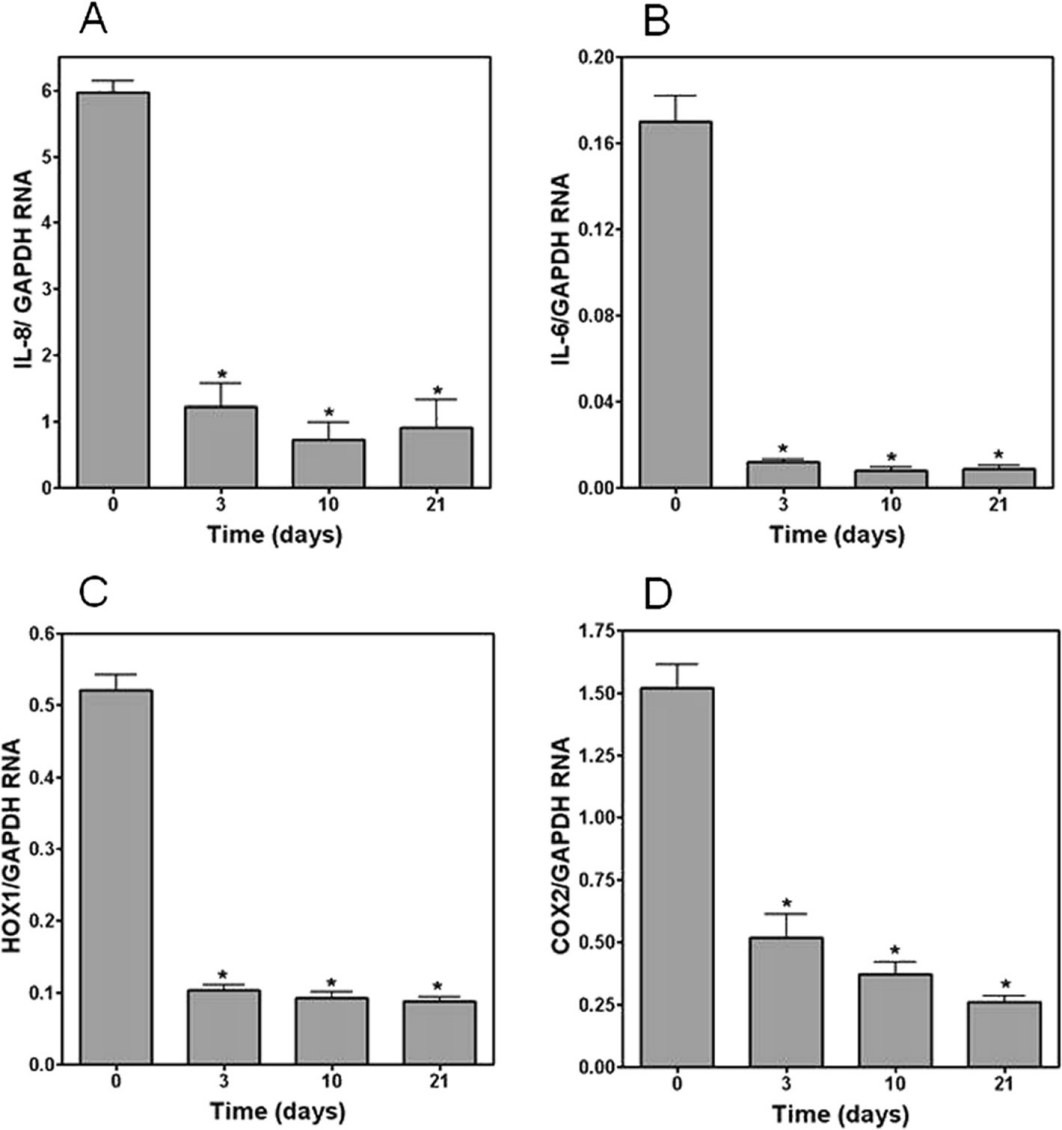
**RT-PCR for IL-8 (A), IL-6 (B), HOX1 (C), and COX2 (D) relative to GAPDH in unexposed NHBE cells.** RNA for all proteins significantly changed with differentiation at ALI. *Significant difference relative to RNA in NHBE cells at day 0.

**Figure 4:**
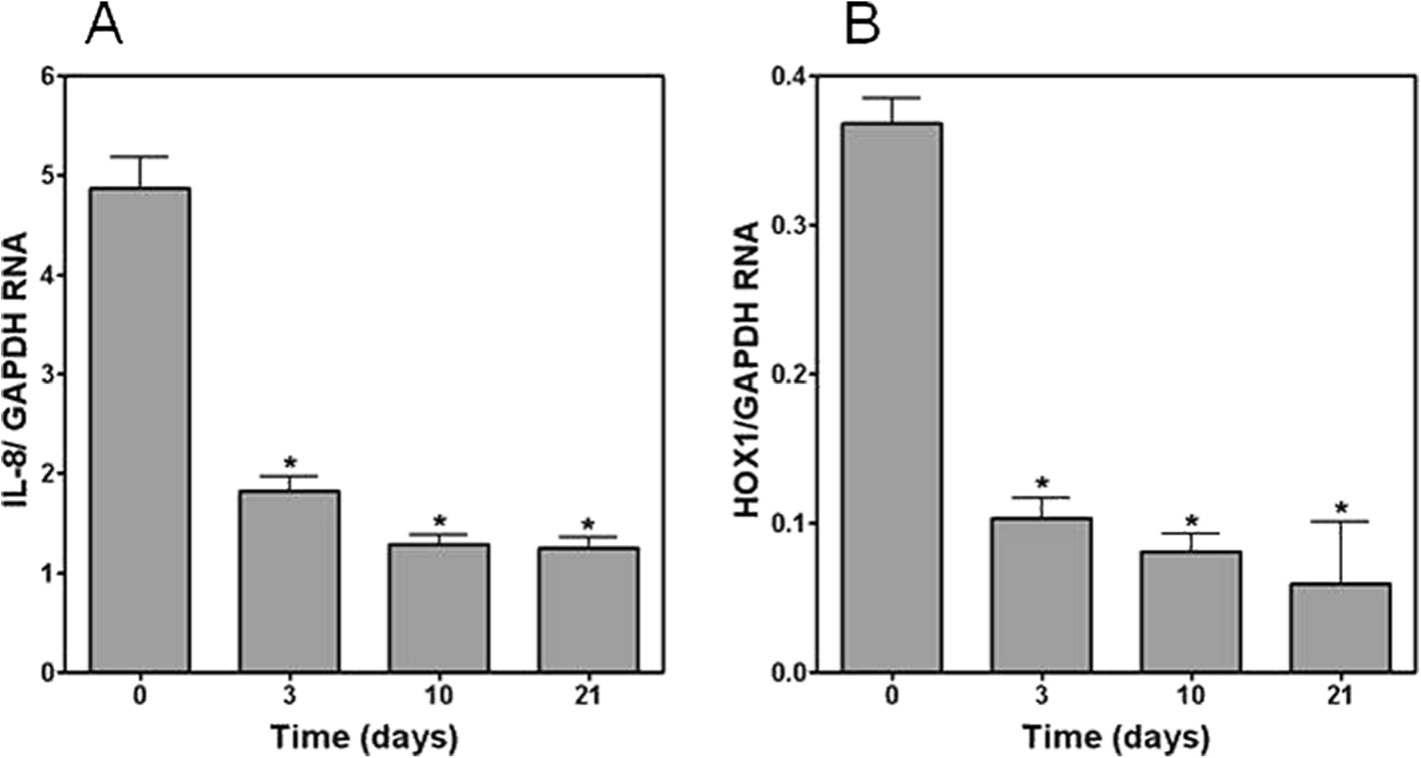
**RT-PCR for IL-8 (A) and HOX1 (B) relative to GAPDH in unexposed BEAS-2B cells.** RNA for both IL-8 and HOX1 significantly decreased with growth at ALI. *Significant difference relative to RNA in BEAS-2B cells at day 0.

The effect of differentiation of NHBE cells grown at ALI on baseline levels of RNA for IL-8, IL-6, HOX1, and COX2 was assessed. Quantities of mRNA for IL-8, IL-6, HOX1, and COX2 all significantly changed during differentiation of NHBE cells at ALI over 3 to 21 days (Figures 3A- 3D). RNA for IL-8, IL-6, HOX1, and COX2 showed rapid changes beginning 3 days after the cells were placed at ALI. BEAS-2B cells grown at ALI also showed changes in RNA for IL-8 and HOX1 which were similar to the NHBE cells with decrements in both between 3 and 21 days of growth at ALI relative to cells grown submerged (Figures 4A and 4B).

**Figure 5:**
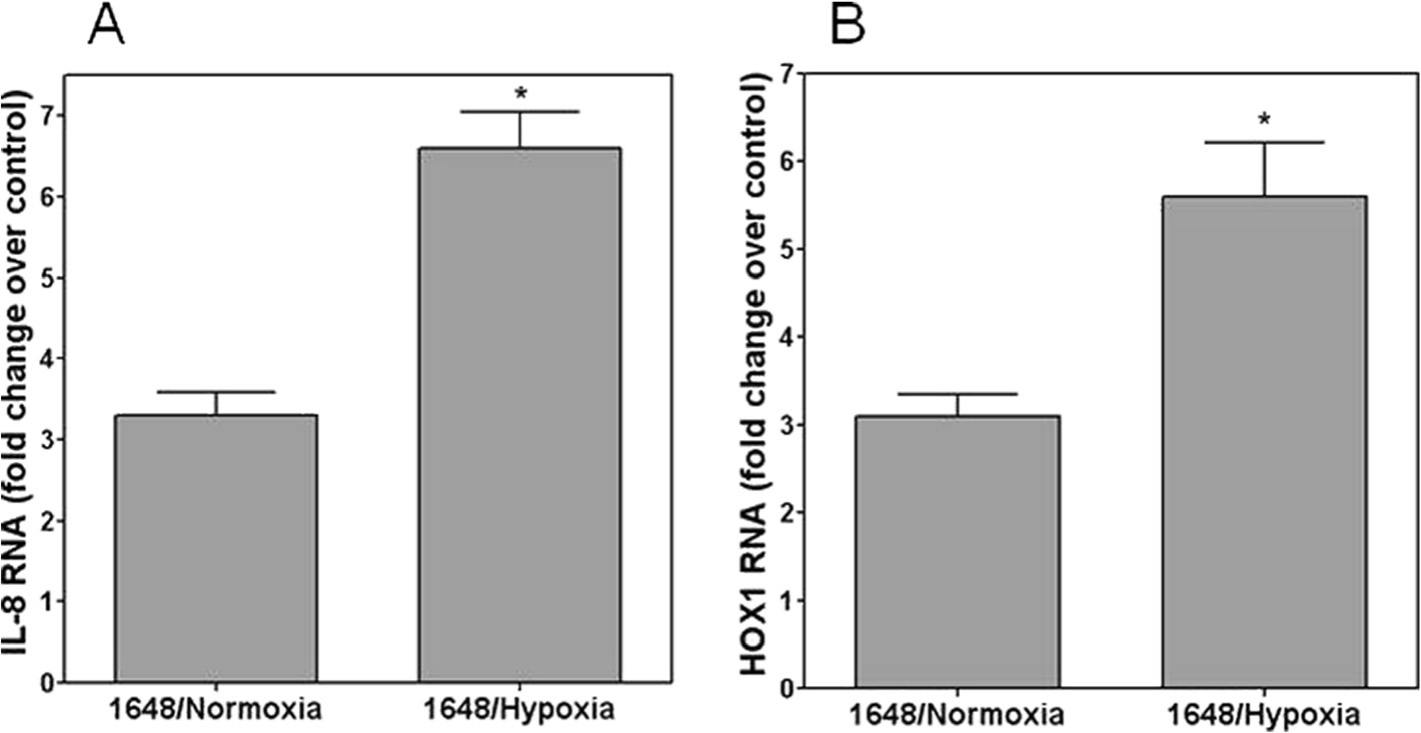
**Fold change RNA of NHBE cells grown in 95% air-5% CO**_**2 **_**(normoxia) and 95% N**_**2**_**-5% CO**_**2 **_**(hypoxia) for 24 hours and exposed to 250 μg NIST 1648 in 25 μL for 4 hr.** The change in RNA for normoxic and hypoxic cells are provided relative to unexposed normoxic and hypoxic cells respectively. While IL-8 and HOX1 RNA was significantly elevated after particle exposure, fold change RNA after hypoxia was significantly increased relative to normoxia. *Significant difference relative to exposed normoxia.

**Figure 6:**
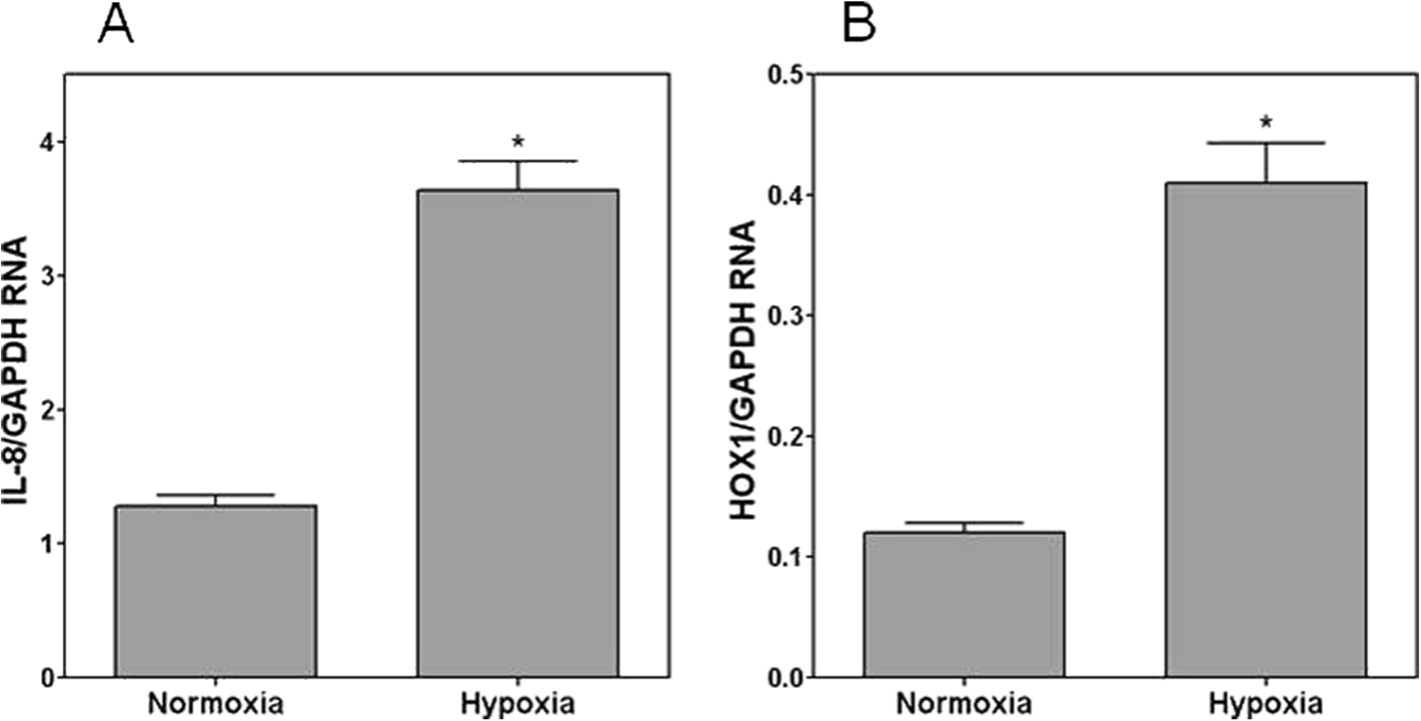
**RT-PCR for IL-8 and HOX1 relative to GAPDH in unexposed NHBE cells grown in 95% air-5% CO**_**2 **_**(normoxia) and 95% N**_**2**_**-5% CO**_**2 **_**(hypoxia) for 24 hours.** RNA for IL-8 and HOX1 significantly increased with hypoxia. *Significant difference relative to normoxia.

Since BEAS-2B cells showed a pattern of response to PM similar to that of NHBE cells (i.e. diminished response to particles when cultured at ALI), it was not possible to attribute these disparities to differentiation alone. Subsequently, another factor was evaluated. Growth at ALI necessitates exposure to an environment which includes higher oxygen availability relative to cells grown submerged in media. To evaluate for an effect of oxygen concentration on response to PM, NHBE cells were grown on transwells in either normoxia or hypoxia for 24 hr and then exposed to 250 μg NIST 1648 in 25 μL. Those cells grown in hypoxia and exposed to the particle showed an approximate 6-fold increase in RNA for IL-8 while those in normoxia had a significantly diminished response (relative to unexposed cells grown in hypoxia and normoxia respectively) (Figure 5A). RNA for HOX1 demonstrated a comparable pattern of response to the particle with elevations in RNA for this protein following exposure in hypoxia and reduced values when the cells were exposed while in normoxia (Figure 5B). IL-8 protein release by cells in hypoxia and normoxia after exposure to the NIST 1648 particle corresponded to RNA changes with cell supernatant concentrations of 654 +/− 79 and 235 +/− 38 pg/mL respectively (with no exposure to PM, the cell release of IL-8 was 25 +/− 7 and 48 +/− 10 pg/mL respectively in hypoxic and normoxic cell supernatant). However, similar to differentiation, placement of the cells in normoxia changed the RNA for both IL-8 and HOX1 with both decreasing (Figures 6A and 6B).

## Discussion

Differentiation of NHBE cells at ALI was associated with a diminished biological response to PM collected from Chapel Hill, North Carolina. Endpoints employed to measure this response included RNA for 1) the pro-inflammatory mediators IL-8 and IL-6 and 2) the proteins HOX1 and COX2 which are involved in numerous forms of oxidative stress to tissues and have been considered both pro-oxidative and anti-oxidative [8-11]. The greatest inflammatory response by NHBE cells appeared to follow exposure of submerged cells to coarse PM. These results support prior investigation which suggested that the coarse fraction of Chapel Hill PM was most closely associated with inflammation and lung injury [12,13]. There were diminished changes in IL-8 and IL-6 RNA following the same exposure of these cells grown on transwells following 3, 10, and 21 days of differentiation. Similarly, changes in HOX1 and COX2 RNA were greatest after exposure of the NHBE cells to the coarse fraction after they were grown submerged. The same pattern of diminished response to PM was observed among BEAS-2B cells grown at ALI relative to submerged conditions. This is an immortalized line of bronchial epithelial cells which does not demonstrate characteristics of cell differentiation such as formation of cilia and mucus secretion. The particle employed in BEAS-2B cell exposures was a NIST reference particle while the endpoints employed included RNA for IL-8 and HOX1. Subsequently, it is not possible to attribute the decrease in PM response to cell differentiation alone since BEAS-2B cells grown at ALI showed the same pattern of response with diminished RNA for IL-8 and HOX1 relative to practice exposure of cells submerged in media. Numerous changes in cell metabolism are likely to be associated with the introduction of ALI (e.g. modifications in those proteins implicated in production and degradation of superoxide and hydrogen peroxide). Over 2500 genes demonstrate significant changes in expression when airway epithelial cells were cultured at ALI; many of these occurred within 48 hours of removal of the media in the apical chamber [6]. Several of these gene products may participate in altering the response to PM. In our investigation, independent of any response to a particle, significant changes in the pro-inflammatory and oxidative baseline mRNA levels were observed in both NHBE cells and BEAS-2B cells with 1) the introduction of ALI and 2) time. RNA for inflammatory mediators were increased on days 3, 10, and 21 in both cell types. Similarly, RNA for HOX1 and COX2 were also altered on days 3, 10, and 21. This suggests that, independent of any exposure, the introduction of ALI and culture of cells over time can be associated with changes in expression of proteins involved in inflammation and oxidative stress.

An obvious disparity between cells grown at ALI and those submerged in medium would be hypoxic conditions associated with the latter. There has been previous investigation demonstrating an interaction between hypoxia and an exposure which augments the pro-inflammatory response in cells. Several of these studies have focused on the interaction between hypoxia and endotoxin. A synergistic effect between *in vitro* exposures to this pro-inflammatory agent and hypoxia in augmenting cellular release of cytokines was observed in cultured microglia and monocytes/macrophages after endotoxin [14,15]. Similarly, *in vivo* exposures (i.e. animal models) to hypoxia significantly increased the response to endotoxin (release of inflammatory mediators and inflammatory cell influx into the lung) [16,17]. The results of our studies using respiratory epithelial cells support a synergistic effect of hypoxia in increasing the pro-inflammatory effect of particle exposure comparable to that described for endotoxin.

Finally, our *in vitro* investigation showed an effect of hypoxia alone in initiating an inflammatory response. Relative to normoxia, RNA for both IL-8, a pro-inflammatory mediator, and HOX-1, a protein of central importance in oxidative stress, increased in NHBE cells exposed to hypoxia. These results are comparable to other studies which demonstrate inflammatory and oxidative effects by hypoxia in both cell and animal models. Inflammatory cytokine secretion, including IL-6 and IL-8, increased several fold in cultured human umbilical vein endothelial cells and macrophages exposed to hypoxia [18,19]. Heme oxygenase was also increased following *in vitro* exposure of both endothelial cells and cardiomyocytes to hypoxia [20,21]. The lungs of rats exposed to hypoxia showed an inflammation with an increased macrophage number as well as elevations in albumin concentration in bronchoalveolar lavage fluid between 1 and 8 hours [22]. Evidence of increased expression in pro-inflammatory mediators, including mRNA for TNF-alpha, macrophage inflammatory protein (MIP)-1beta, and monocyte chemoattractant protein (MCP)-1, were detected in lavaged macrophages exposed to hypoxia.

## Conclusion

We conclude that growth of airway epithelial cells at ALI is associated with a diminished biological effect following exposure to particles. This lack of response was not related to differentiation alone but also likely reflected increased oxygen availability in cells cultured over time at ALI.

## Materials and methods

### Cell cultures

The protocol for the acquisition of NHBE cells was approved by the University of North Carolina School of Medicine Committee on Protection of the Rights of Human Subjects and by the U.S. EPA. Subjects were informed of the procedures and potential risks and each signed an informed consent. NHBE cells were obtained from healthy individuals through bronchoscopy with bronchial brushings. Cells were expanded to passage-3 in bronchial epithelial growth medium (BEGM; Clonetics, San Diego, CA), plated on collagen-coated filters with a 0.4-micron pore size (Trans-CLR, Costar, Cambridge, MA) at a density of 1 × 105 cells/filter and inserted into 12 well culture plates [6,23]. Cells were maintained in a 1:1 mixture of BEBM and Dulbecco’s Modified Eagles Medium (DMEM) with high glucose, growth supplements, bovine pituitary extracts, bovine serum albumin, and nystatin. Fresh medium (0.5 mL in the apical chamber and 1.0 mL in the basal chamber) was provided every 48 hours. In those cells grown at ALI, the apical medium was removed (day 0). Submerged cells were continued with 0.5 mL in the apical chamber. With confluence of the cells, retinoic acid was added to the media to promote differentiation in cells grown both at ALI; submerged cells also received this supplement. The cells were maintained for 21 days allowing those grown at ALI to differentiate into ciliated, mucus-producing cells (occurring at approximately day 10 and later). Fresh medium was provided every 48 hours (1.0 mL in the basal chamber for those grown at ALI and 0.5 mL and 1.0 mL in the apical and basal chambers for those grown submerged).

BEAS-2B cells were also used in *in vitro* studies. This is an immortalized line of human bronchial epithelial cells derived by transfection of primary cells with SV40 early-region genes. These cells have not been reported to undergo differentiation and cells similarly transformed with SV40 oncogene have not demonstrated such a capacity [24]. Comparable to the NHBE cells, BEAS-2B cells were plated on the same collagen-coated filters and inserted into 12 well culture plates. The cells were maintained in keratinocyte growth medium (KGM; Clonetics) which is essentially MCDB 153 medium with supplemented human epidermal growth factor, insulin, hydrocortisone, calcium, bovine pituitary extract, ethanolamine and phosphoethanolamine. Fresh medium (0.5 mL in the apical chamber and 1.0 mL in the basal chamber) was provided every 48 hours. Following confluence of the cells, ALI was created on day 0 by removing the apical medium. The cells were maintained for 21 days after this. Fresh medium (1.0 mL in the basal chamber) was provided every 48 hours.

### Particle exposure

NHBE cells were exposed to coarse, fine, and ultrafine fractions of ambient air pollution particle. Particles were collected outside the U.S. Environmental Protection Agency (EPA) Human Studies Facility in Chapel Hill, North Carolina using a ChemVol model 2400 high volume cascade impactor (Rupprecht & Patashnick Co., Albany, NY) [25]. Coarse PM (PM_2.5–10_ μm) and fine PM (PM_0.1–2.5_ μm) were collected onto polyurethane foam (McMaster-Carr, Atlanta, GA), which was previously cleaned with methanol and water and dried under sterile conditions. Ultrafine particles (PM < 0.1 μm) were collected onto G5300 filters (Monandock Non-Wovens LLC, Mt. Pocono, PA). The foam or filter was pre-wetted with a small amount of 70% ethanol, and endotoxin-free water was added to a total volume of 40 mL. The particles were removed from the foam or filter by sonication for 1 hour in a water bath (FS220; Fisher Scientific, Pittsburgh, PA). The foam was removed and particles were then lyophilized.

Cultured BEAS-2B cells were exposed to NIST 1648 (National Institute of Standards and Technology; Gaithersburg, MD). This was an ambient air pollution particle collected in St. Louis, Missouri and has been previously characterized [26].

Immediately prior to exposure to particles, the apical chamber of the cultured cells was washed with 500 μL PBS and the buffer immediately removed. Particle in 25 μL PBS was placed on the cells in the apical chamber of the transwells and agitated on a rocking shaker (Reliable Scientific, Nesbit, MS) at an intermediate speed for a minimum of 2 minutes. Dispersion was documented by visual inspection of the cells using an inverted microscope at a magnification of 100×. Particle exposure continued until collection of the specific endpoint. For RT-PCR, this was 4 hours while for cytokines this was 24 hours. Cytotoxicity, assessed using release of LDH after exposure to particles, was demonstrated to be insignificant. The response of submerged cells of a specific age exposed to particle was compared to submerged cells of that same age without exposure and the response of cells grown at ALI to a specific age exposed to particle was compared to these cells grown at ALI of that same age without exposure.

### RT-PCR

Relative gene expression in NHBE and BEAS-2B cells was quantified using real-time quantitative PCR. RNA was reverse transcribed to generate cDNA. Primer/probe sets were obtained as Taqman pre-developed assay reagents (concentrated and pre-optimized mix of primers and FAM-labeled Taqman probe) from Applied Biosystems (University Park, IL). Quantitative fluorogenic amplification of cDNA was performed using the ABI Prism 7500 Sequence Detection System (Applied Biosystems) primer/probe sets of interest, and TaqMan Universal PCR Master Mix (Applied Biosystems). The relative abundance of mRNA levels was determined from standard curves generated from a serially diluted standard pool of cDNA prepared from cultured respiratory epithelial cells. The relative abundance of GAPDH mRNA was used to normalize levels of the mRNAs of interest. Specific proteins for which RNA was measured included two involved in differentiation (alpha tubulin and muc5B), two involved in inflammation (interleukin-8 and interleukin-6; IL-8 and IL-6 respectively), and two involved in oxidative stress (heme oxygenase 1 and cyclooxygenase 2; HOX1 and COX2 respectively).

### Cell release of interleukin (IL)-8 and IL-6

Cells were exposed to particle for 24 hr. IL-8 and IL-6 concentrations in the cell media were measured using commercially available ELISA kits (R&D Systems, Minneapolis, MN).

### Cell air and hypoxia exposures

Two modular chambers (Billups-Rothenberg, Modular Incubator Chamber, Del Mar, CA) were placed in an incubator, set at 37°C, and each was ventilated with either 95% air-5% CO_2_ (normoxia) or 95% N_2_-5% CO_2_ (hypoxia). Humidity was supported with the inclusion of a petri dish of deionized water included in the chambers. The flow to each chamber (approximately 2 L/min) was adjusted so that there was constant exchange of the gas mixtures. The percentage oxygen in the two chambers was monitored using Handi + analyzers (Maxtec, Salt Lake City, UT).

NHBE cells were employed during investigation of the effect of hypoxia on the response to particle. Cells were grown in 12 well culture plates, maintained in KGM (0.5 mL in the apical chamber and 1.0 mL in the basal chamber), and allowed to grow to confluence. The apical medium was removed and the cells immediately placed in either normoxia or hypoxia for 24 hr. At 24 hrs, either 25 μL PBS or NIST 1648 particle in 25 μL PBS was placed on the apical aspect of the cells.

### Statistics

The minimum number of replicates for all measurements was nine; with NHBE cells, this included three different volunteers’ cells. Data are expressed as mean value ± standard error. Differences between multiple groups were compared using one-way analysis of variance. The post-hoc test employed was Scheffe’s test. Differences between two groups were compared using T tests of independent means. Two-tailed tests of significance were employed. Significance was assumed at P < 0.05.

## Competing interests

The authors declare that they have no competing interests.

## Authors’ contributions

All authors performed experiments, analyzed data, drafted the manuscript, and approved the final manuscript.
